# Counter Practice in France

**Published:** 1879-04

**Authors:** 


Counter Practice in France.—The law against the illegal exercise of medicine in France is very severe, but if it were generally enforced pharmacy would be impossible. Lately a man had a slight burn, and his wife fetched from a pharmacien a few sous' worth of "eau blanche," or lead lotion. The man died a few days after, according to the testimony of the physician who attended him, of tetanus. The Government medical officer who had to register the death, reported the cause of death to have been cerebral hemorrhage. The widow, however, took the fancy that it was the lotion of the pharmacien which had killed her husband, and she consequently laid a charge against him. The lotion was analyzed and the case came on. The evidence was too weak to convict the poor pharmacien of manslaughter, but the court fined him 625f. for the illegal exercise of pharmacy!—*Chemist and Druggist.*

